# The development and application of a molecular community profiling strategy to identify polymicrobial bacterial DNA in the whole blood of septic patients

**DOI:** 10.1186/s12866-015-0557-7

**Published:** 2015-10-16

**Authors:** MMP Faria, JM Conly, MG Surette

**Affiliations:** Department of Microbiology, Immunology and Infectious Diseases, University of Calgary, Calgary, AB T2N 4 N1 Canada; Department of Medicine, University of Calgary, Calgary, AB T2N 4 N1 Canada; Department of Pathology and Laboratory Medicine, University of Calgary, Calgary, AB T2N 4 N1 Canada; Department of Calvin, Phoebe and Joan Snyder Institute for Chronic Diseases, University of Calgary, Calgary, AB T2N 4 N1 Canada; Farncombe Family Digestive Health Research Institute, Departments of Medicine and Biochemistry and Biomedical Sciences, Faculty of Health Sciences, McMaster University, 1280 Main Street, HSC 3 N 8 F, Hamilton, ON L8S 4 K1 Canada; Department of Medicine, McMaster University, Hamilton, ON L8S 4 K1 Canada; Biochemistry and Biomedical Sciences, McMaster University, Hamilton, ON L8S 4 K1 Canada

**Keywords:** Sepsis, Saponin, Blood, 16 s rRNA, Illumina, Polymicrobial, DNA Sequencing

## Abstract

**Background:**

The application of molecular based diagnostics in sepsis has had limited success to date. Molecular community profiling methods have indicated that polymicrobial infections are more common than suggested by standard clinical culture. A molecular profiling approach was developed to investigate the propensity for polymicrobial infections in patients predicted to have bacterial sepsis.

**Results:**

Disruption of blood cells with saponin and hypotonic shock enabled the recovery of microbial cells with no significant changes in microbial growth when compared to CFU/ml values immediately prior to the addition of saponin. DNA extraction included a cell-wall digestion step with both lysozyme and mutanolysin, which increased the recovery of terminal restriction fragments by 2.4 fold from diverse organisms. Efficiencies of recovery and limits of detection using Illumina sequencing of the 16S rRNA V3 region were determined for both viable cells and DNA using mock bacterial communities inoculated into whole blood. Bacteria from pre-defined communities could be recovered following lysis and removal of host cells with > 97 % recovery of total DNA present. Applying the molecular profiling methodology to three septic patients in the intensive care unit revealed microbial DNA from blood had consistent alignment with cultured organisms from the primary infection site providing evidence for a bloodstream infection in the absence of a clinical lab positive blood culture result in two of the three cases. In addition, the molecular profiling indicated greater diversity was present in the primary infection sample when compared to clinical diagnostic culture.

**Conclusions:**

A method for analyzing bacterial DNA from whole blood was developed in order to characterize the bacterial DNA profile of sepsis infections. Preliminary results indicated that sepsis infections were polymicrobial in nature with the bacterial DNA recovered suggesting a more complex etiology when compared to blood culture data.

## Background

Sepsis is defined based on the clinical symptoms and signs of a systemic inflammatory response due to a microbial infection in sterile body sites or fluids [1]. Despite modern technologies and advances in health care, sepsis rates continue to climb and have more than doubled in the last ten years [2]. The socioeconomic costs associated with sepsis are high due to the increased need for hospital resources and long stays in the intensive care unit (ICU) [2]. In Canada, sepsis is one of the leading causes of in-hospital mortality with 10.9 % of hospital deaths in 2008–2009 being sepsis related [2]. Bacteria are implicated in the majority of sepsis infections with three independent studies reporting bacteremia, confirmed or suspected, in 63–74 % of all cases [3–5]. Currently, the “gold standard” of sepsis diagnostics is a confirmed bloodstream infection using blood culture. Microbial growth is monitored continuously and is detected using gas production. In order to identify the microbial growth, sub-culturing and biochemical testing is performed using clinical and laboratory standards institute (CLSI) guidelines [6]. The use of this diagnostic tool is time consuming and lacks sensitivity due to several inherent limitations such as a dependency on volume with efficacy decreasing as volume decreases, limited efficacy for slow-growing or fastidious organisms, and incubation times of 72–96 h [7]. These limitations allow an overall positivity of blood culture in the range of 30–40 % [7]. Since survival rates have been reported to decline with every hour without therapy, clinicians usually treat with broad-spectrum empiric antibiotics and then use any blood culture results obtained to tailor antimicrobial therapy [8].

There is a need for more rapid and comprehensive diagnostics for bloodstream infections in the management of sepsis. Nucleic acid technologies and proteomic approaches are being actively explored for sepsis diagnostics. Molecular testing on whole blood has capitalized on developments in PCR-based technology [9]. Broad-range assays, with primers targeting variable regions in the 16S rRNA or 18S/23S rRNA gene, have the greatest clinical applicability for sepsis diagnostics due to their short turnaround time and ability to directly detect any non-cultivable or cultivable pathogens [6, 9, 10].

To date, the application of nucleic acid technology in sepsis has focused on identifying 1–2 organisms from positive blood culture [11–16]. With the advent of next-generation sequencing technologies, there has been a shift in focus from singular pathogens causing illness to the concept of polymicrobial communities of organisms, pathogens and commensals in human infections. Despite this, the concept of a sepsis microbiota has not been explored and sepsis infections are considered to be monomicrobial with polymicrobial infections occurring in a minority of patients [17]. In order to better understand bloodstream infections in sepsis, we developed a method to optimize recovery microbial cells and DNA directly from blood in order to assess the bacterial DNA profile in sepsis patients using molecular community profiling methods of terminal restriction fragment length polymorphism (TRFLP) and paired-end Illumina sequencing. In this paper we outline the methods developed, their success in mock community studies and application to sepsis case studies.

## Results

### Impact of saponin on bacterial isolate viability

Whole blood is known to contain many substituents that can impair PCR including heme, leukocyte DNA, immunoglobulin G, hemoglobin and lactoferrin [18]. In this study, a method of lysing blood cells prior to DNA extraction was evaluated in order to improve the recovery of bacterial cells and DNA from small volumes of blood. Saponin, a plant metabolite, is known for its ability to lyse both red and white blood cells [19, 20]. Recent studies have shown that saponin at low concentrations (1 % w/v) does not impact microorganism growth [20–22]. In order to confirm the results from these studies, the ability of bacteria to grow directly in saponin culture was assessed.

**Fig. 1:**
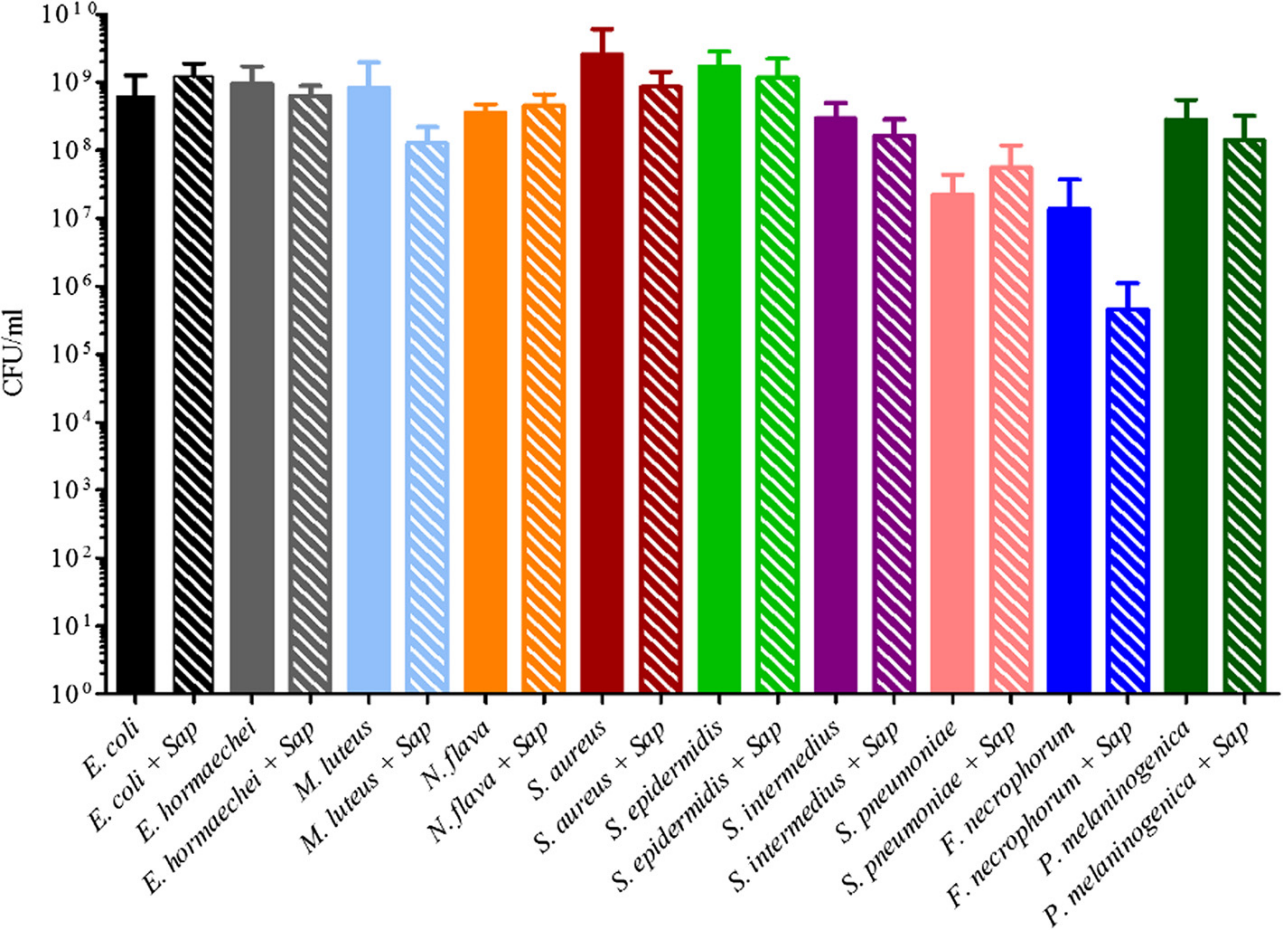
The addition of 0.85 % saponin to bacterial cells does not impact viability. The average CFU/ml and the standard deviation were plotted for each sample prior to and after the incubation with 0.85 % saponin (represented by “sap” in the figure label) for 1 h at room temperature either with ambient air or in an anaerobic chamber for *F. necrophorum* and *P. melaninogenica.* Each experiment was done in triplicate with samples recorded in duplicate. There were no statistically significant differences found in the viable cell count of the bacteria (two-tailed Students *t*-test, *p* > 0.05 % significance)

Following incubation with the strains recovered from sepsis infections (Table 1), there were no significant changes in microbial growth when compared to CFU/ml values immediately prior to the addition of saponin (Fig. 1). Only *Fusobacterium necrophorum* had a 2-log drop in CFU/ml following treatment with saponin but the reduction was not significant (*p* = 0.2375, two-tailed *t*-test). The observed decrease in viability of *F. necrophorum* may result from its sensitivity to oxygen. Although incubations were carried out under anaerobic conditions, initial sample preparations were carried out with some exposure to ambient oxygen. These results demonstrated that bacteria were not affected by the presence of saponin.

**Table 1:** Isolates used in synthetic community assessments

Species	Source	Ward	Growth Conditions	Media
*Enterobacter hormaechei*	Peritoneal Fluid	FMC ICU	5 % CO_2_	CBA
*Escherichia coli*	Urine	FMC ICU	5 % CO_2_	MAC
*Fusobacterium necrophorum*	BAL	FMC ICU	Anaerobic	FAA
*Micrococcus luteus*	Whole blood	FMC ICU	5 % CO_2_	TSY
*Neisseria flava*	BAL	FMC ICU	5 % CO_2_	TSY
*Prevotella melaninogenica*	BAL	FMC ICU	Anaerobic	FAA
*Staphylococcus aureus*	Whole blood	FMC ED	5 % CO_2_	MSA
*Staphylococcus epidermidis*	Whole blood	FMC ICU	5 % CO_2_	MSA
*Streptococcus intermedius*	Abscess fluid	FMC ICU	5 % CO_2_	CNA
*Streptococcus pneumoniae*	Chest tube aspirate	FMC ICU	5 % CO_2_	CNA

### Optimization of DNA extraction from whole blood using enzymatic lysis

**Fig. 2:**
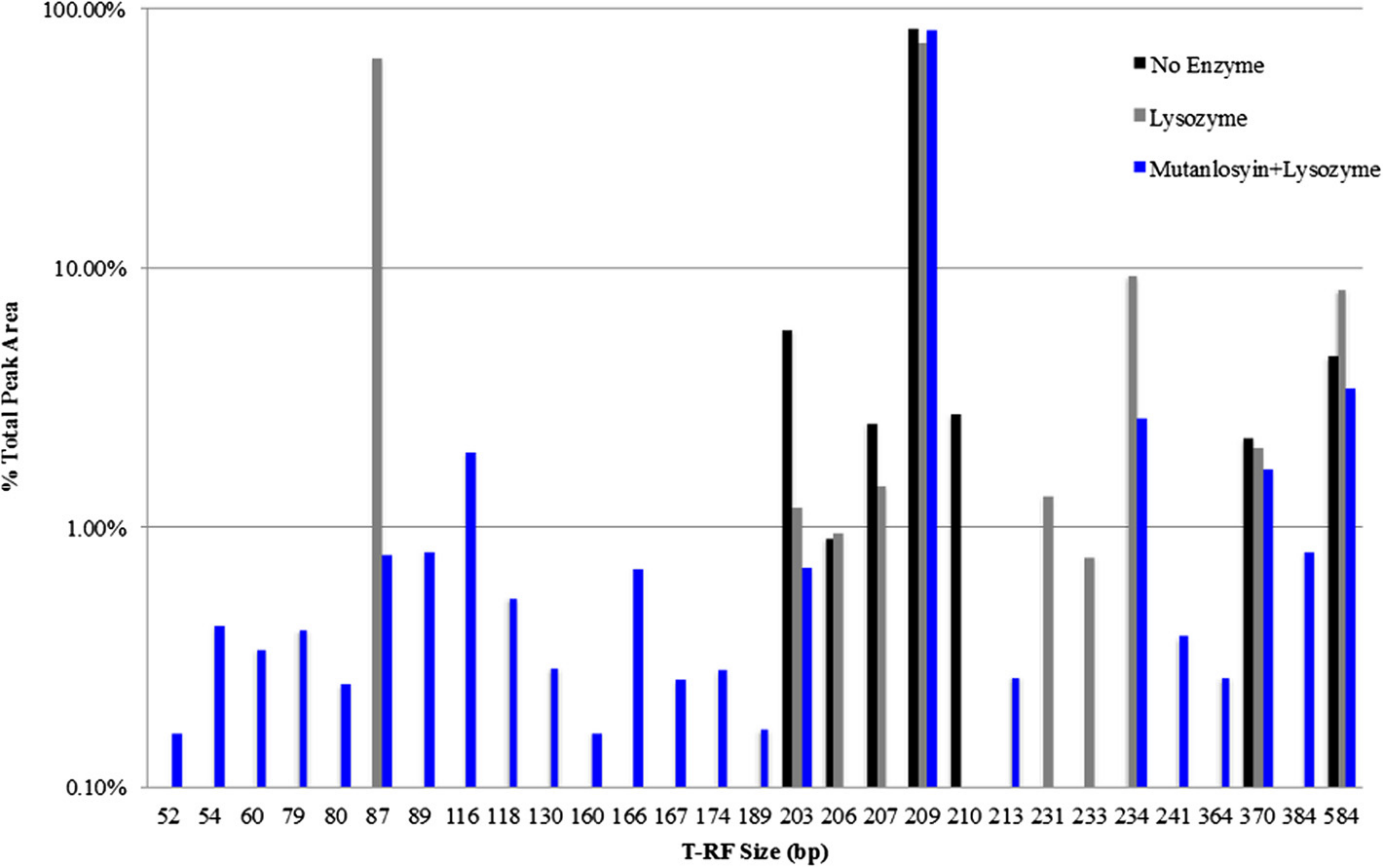
Enzymatic digestion requirement for bacterial DNA extraction of whole blood. Whole blood was spiked with a CBA plate pool from ASN087 BAL sample. Following a treatment with saponin, the resulting pellet was either digested with no enzyme (*black*), lysozyme (*grey*), or lysozyme and mutanolysin (*blue*). More T-RFs were present when enzymatic digestion was done with lysozyme (10, *grey*) and lysozyme plus mutanolysin (24, *blue*) identified, respectively, when compared to no enzymatic treatment with only 7 T-RFs present

Our standard lab protocol included enzymatic digestion of the bacterial cell wall in a pretreatment step [23–25]. To evaluate if this was necessary in samples recovered from blood, a microbial community made from pooling all bacteria present on a Columbia Blood Agar (CBA) plate inoculated with bronchoalveolar lavage (BAL) fluid from a septic patient (ASN087) was spiked into blood and the bacterial DNA recovered using variations of our protocol were analyzed by terminal restriction fragment length polymorphism (TRFLP). Table 2 indicates the organisms represented in the culture pool. TRFLP analysis of the DNA recovered following treatment with no bacterial cell wall digesting enzymes had 7 principal T-RF peaks recovered (Fig. 2). The addition of lysozyme increased the recovery to 10 T-RFs whereas digestion with lysozyme and mutanolysin increased recovery to 24 T-RFs (Fig. 2). Based on this data, the DNA extraction method developed for use with whole blood samples included a cell-wall digestion step with both lysozyme and mutanolysin. Given the limitations of TRFLP in terms of providing a semi-quantitative assessment of highly concentrated culture pools it was only used for the initial method development. Further analysis was done using Illumina sequencing of PCR amplified 16S rDNA.

**Table 2:** Organisms recovered from ASN087 BAL fluid cultivated on CBA and their respective T-RF cut size

16S Identification (HOMD)	T-RF Size (bp)	CFU/ml
*Capnocytophaga sp*	86	10^2^
*Prevotella melaninogenica*	99	10^5^
*Lachnospiraceae [G-1] sp*	108	10^4^
*Fusobacterium necrophorum*	194	10^5^
*Bifidobacteriaceae [G-1] sp*	202	10^6^
*Neisseria flava*	209	10^6^
*Staphylococcus epidermidis*	234	10^3^
*Neisseria flavescens*	370	10^4^
*Neisseria elongata*	370	10^4^
*Streptococcus mitis*	574	10^3^
*Capnocytophaga sp*	584	10^4^
*Streptococcus anginosus*	584	10^4^
*Streptococcus constellatus*	584	10^5^

### Saponin treatment of blood spiked with synthetic communities

**Fig. 3:**
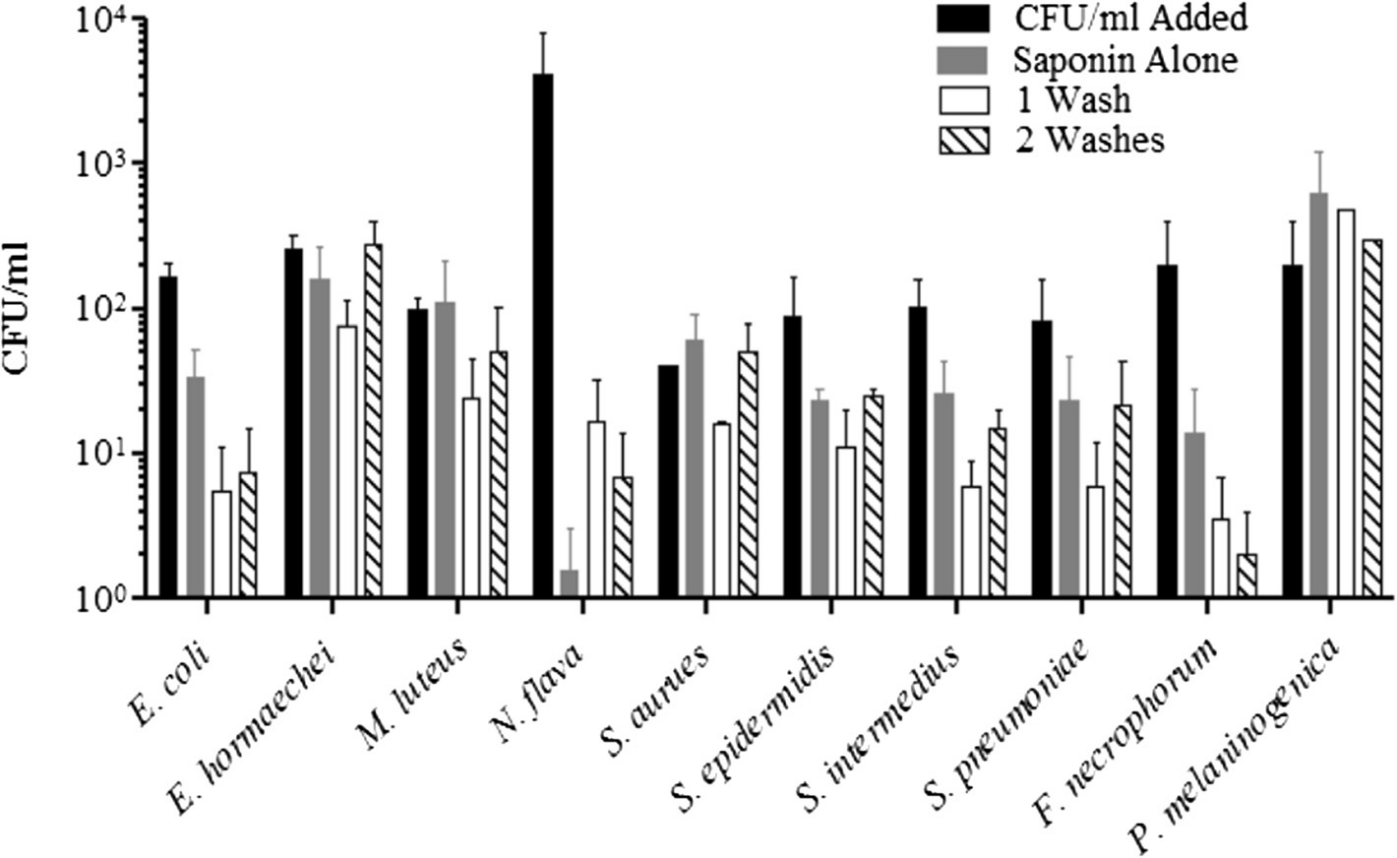
Limit of detection for synthetic communities of bacteria spiked into whole blood. The CFU/ml of each bacterium in the community was determined prior to blood spiking (*solid black bars*). The CFU/ml of bacteria recovered after each step in the saponin-blood treatment protocol was determined; addition of 0.85 % saponin with no further washes (*solid grey bars*), addition of saponin at 0.85 % with the addition of a 1 ml sterile double distilled water wash (*diagonal lined bars*), or addition of saponin at 0.85 % with two washes with 1 ml sterile double distilled water (*solid white bars*). Each organism was spiked into whole blood alone to verify the limit of detection observed in Table 3 (D)

In order to determine if our method could recover total microbial abundance from a defined community, a mock community of organisms typically recovered from sepsis infections was used. The applicability of the saponin blood-treatment method was assessed in both cultivation-dependent and -independent approaches. The mock community was spiked into blood collected from healthy donors in vacutainers to parallel the collection from actual septic patients enrolled in the study. The addition of saponin to lyse red blood cells and washing to remove debris from the lysis of the human cells on bacterial DNA recovery of the synthetic community (Table 1) was examined at different bacterial concentrations. 16S rRNA community profiling as described was used to assess recovery. The limit of detection and percent recovery is summarized in Table 3. Following the addition of saponin and no washes, the percent recovery of synthetic community organisms was above 80 % for 9 out of the 10 organisms in SC1 and SC3 but only 4 out of 10 for SC5 (Table 3). The treatment with saponin and one wash had highly variable results with percent recovery above 80 % for 8 of the 10 organisms in SC1, 6 out of 10 for SC3 and 1 out 10 for SC5 (Table 3). After two washes, 5 of the 10 organisms could be recovered from all SCs regardless of concentration (Table 3). In order to examine the effects of competition in the recovery of the bacteria, each organism was spiked into whole blood independently. Recovery of each organism was optimal using saponin alone with the notable exception of *N. flava* (Fig. 3). Anaerobic organisms, *F. necrophorum* and *P. melaninogenica*, were recovered under all treatment conditions when cultivated alone and under anaerobic conditions (Fig. 3).

**Table 3:** Percent recovery and limit of detection for mock community spiked into whole blood

	Percent recovery (%)									Limit of detection (CFU/ml)		
	Saponin alone			Saponin + 1 Wash			Saponin + 2 washes			Saponin alone	Saponin +1 wash	Saponin + 2 washes
Organism	SC1	SC3	SC5	SC1	SC3	SC5	SC1	SC3	SC5			
*E. coli*	82.8	100	73.7	24.8	0	0	32.3	51.7	15.5	10^2^	10^4^	10^3^
*E. hormaechei*	100	100	100	100	100	100	100	100	100	10	10	10
*N. flava*	100	100	23.4	100	100	75.6	100	100	37.8	10^5^	10^4^	10^4^
*M. luteus*	100	68.7	8.0	100	6.9	0	20.1	0	0	10^2^	10	10
*S. epidermidis*	100	100	100	100	100	39.6	100	100	100	10	10	10
*S. aureus*	100	100	39.3	100	100	31.7	100	100	100	10	10	10
*S. intermedius*	100	100	100	100	100	19.9	100	100	100	10	10	10
*S. pneumoniae*	100	100	0	96.9	100	0	100	100	100	10^2^	10^2^	10
*F. necrophorum* ^a^	0	100	0	0	0	0	0	0	0	10^2^	10^3^	10^4^
*P. melaninogenica* ^a^	100	100	0	100	0	0	0	0	0	10^2^	10^2^	10^2^

^a^Based on recovery alone (Fig. 3) not in SC

**Fig. 4:**
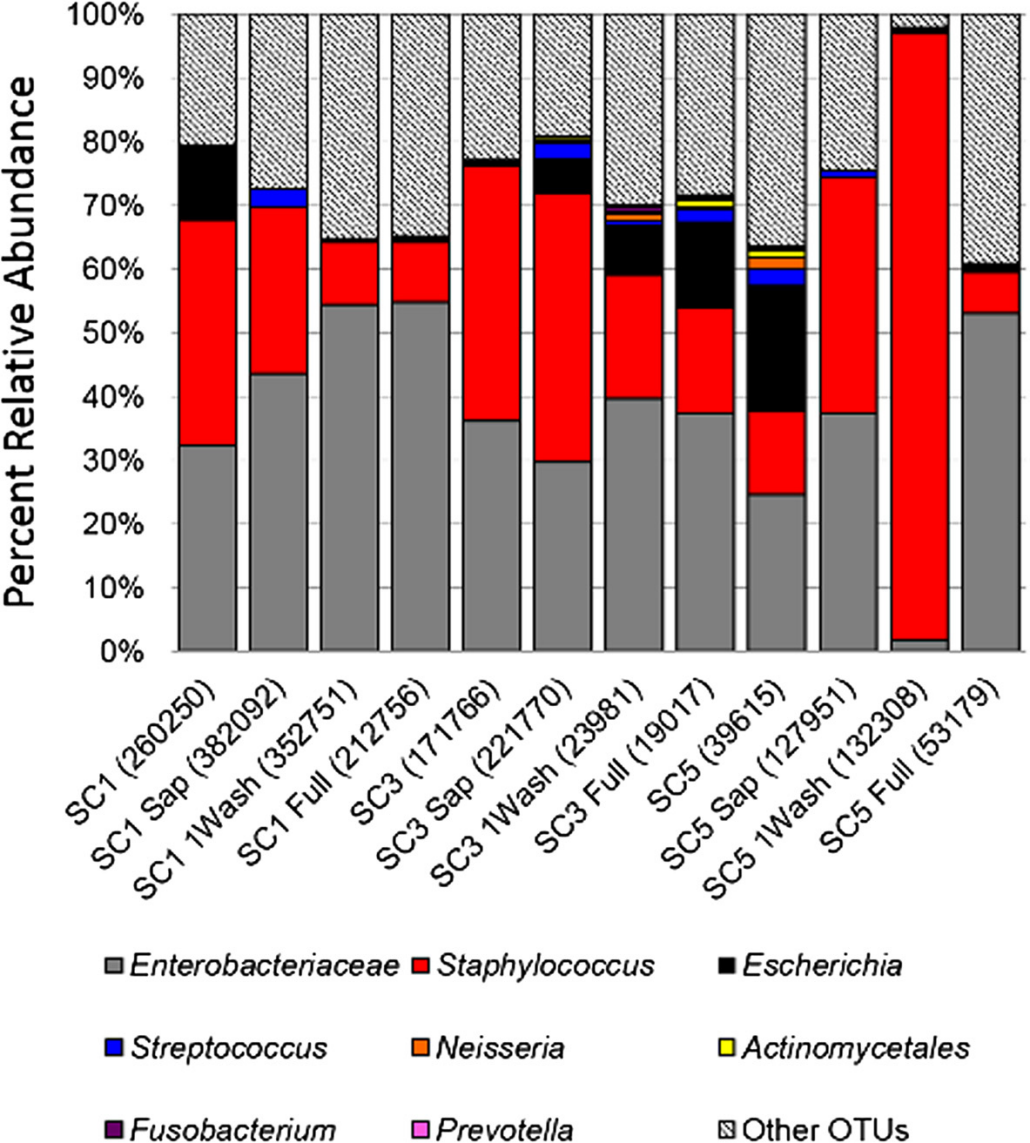
OTU abundance of 16S rRNA Illumina sequenced DNA from synthetic communities. Taxonomic summaries for the synthetic community samples after each step in the saponin blood-treatment protocol were compared. Each bar represents the total PCR amplified DNA sequenced for the sample and the relative abundance of each OTU in the molecular profile. The representative sequence for each OTU was aligned to both the NCBI and HOMD 16S databases in order to determine what synthetic community organism they represented. *E. hormaechei* sequences were represented by the *Enterobacteriaceae* OTU, *S. aureus* was represented by the *Staphylococcus* OTU, *K. pneumoniae* by the *Klebsiella* OTU, *E. coli* by the *Escherichia* OTU, *S. pneumoniae* and *S. intermedius* by the *Streptococcus* OTU, *N. flava* by the *Neisseria* OTU, *M. luteus* by the *Actinomycetales* OTU, *F. necrophorum* by the *Fusobacterium* OTU, and *P. melaninogenica* by the *Prevotella* OTU. All OTUs with sequence alignments that could not be correlated to the bacteria spiked into the synthetic community were combined into “Other OTUs”, which accounted for 20–40 % of the total OTU abundance

Bacterial DNA extracted from each community prior to treatment and after all blood spiking conditions was analyzed. Sequence reads that did not map to the bacterial 16S rRNA reference library resulting from primer cross-reactivity to human DNA [26, 27] were removed. A total of 210 OTUs were identified. OTUs were filtered to remove OTUs representing less than 0.1 % total abundance, resulting in 32 OTUs representing 97 % or greater of the total amplified bacterial DNA present. The top 3 OTUs in all samples were those representing the genera *Streptococcus, Staphylococcus,* and *Enterobacter* (Fig. 4). The family *Enterobacteriaceae* OTU was also prevalent in all samples (Fig. 4). *Micrococcus* DNA could only be identified at the order level, *Actinomycetales* (Fig. 4). DNA from 9 of the 10 community organisms was recovered from all treatments with *Prevotella, Micrococcus* and *Neisseria* OTUs present at scant levels (Table 4). *Fusobacterium* DNA could not be recovered from SC5 when treated with saponin and with saponin plus two washes (Table 4). Other DNA represented OTUs that did not match DNA from the organisms in the synthetic community. It was present from 0.1 % in the SC1 communities up to 8 % in the SC5 communities (Table 4).

**Table 4:** Relative abundance of OTUs recovered from synthetic communities spiked into whole blood

OTU taxonomic ID	SC1	SC1 Sap	SC1 1Wash	SC1 Full	SC3	SC3 Sap	SC3 1Wash	SC3 Full	SC5	SC5 Sap	SC5 1Wash	SC5 Full
*Enterobacteriaceae* (*E. hormaechei*)	32.22 %	43.58 %	54.37 %	54.78 %	36.15 %	29.76 %	39.65 %	37.26 %	24.60 %	37.40 %	1.94 %	53.01 %
*Staphylococcus* (*S. aureus*, *S. epidermidis*)	35.59 %	26.11 %	9.76 %	9.40 %	40.03 %	42.14 %	19.45 %	16.69 %	13.03 %	36.95 %	94.93 %	6.54 %
*Escherichia* (*E. coli)*	10.82 %	0.01 %	0.33 %	0.26 %	0.90 %	5.36 %	7.56 %	13.30 %	19.67 %	0.03 %	0.70 %	1.00 %
*Streptococcus* (*S. pneumoniae*, *S. intermedius*)	0.30 %	2.98 %	0.17 %	0.24 %	0.00 %	2.56 %	0.80 %	2.08 %	2.72 %	1.27 %	0.16 %	0.19 %
*Neisseria* (*N. flava*)	0.13 %	0.00 %	0.06 %	0.10 %	0.00 %	0.24 %	1.20 %	0.36 %	1.83 %	0.00 %	0.03 %	0.01 %
*Actinomycetales* (*M. luteus*)	0.01 %	0.01 %	0.02 %	0.12 %	0.00 %	0.55 %	0.28 %	1.09 %	0.95 %	0.00 %	0.12 %	0.07 %
*Fusobacterium *(*F. necrophorum*)	0.12 %	0.00 %	0.05 %	0.13 %	0.00 %	0.29 %	0.75 %	0.40 %	0.40 %	0.00 %	0.02 %	0.00 %
*Prevotella* (*P. melaninogenica*)	0.25 %	0.00 %	0.01 %	0.14 %	0.00 %	0.02 %	0.33 %	0.44 %	0.31 %	0.01 %	0.01 %	0.02 %
Other OTUs	20.56 %	27.32 %	35.23 %	34.84 %	22.90 %	19.08 %	29.98 %	28.39 %	36.47 %	24.34 %	2.09 %	39.15 %

### DNA profiling of healthy donor blood

**Fig. 5:**
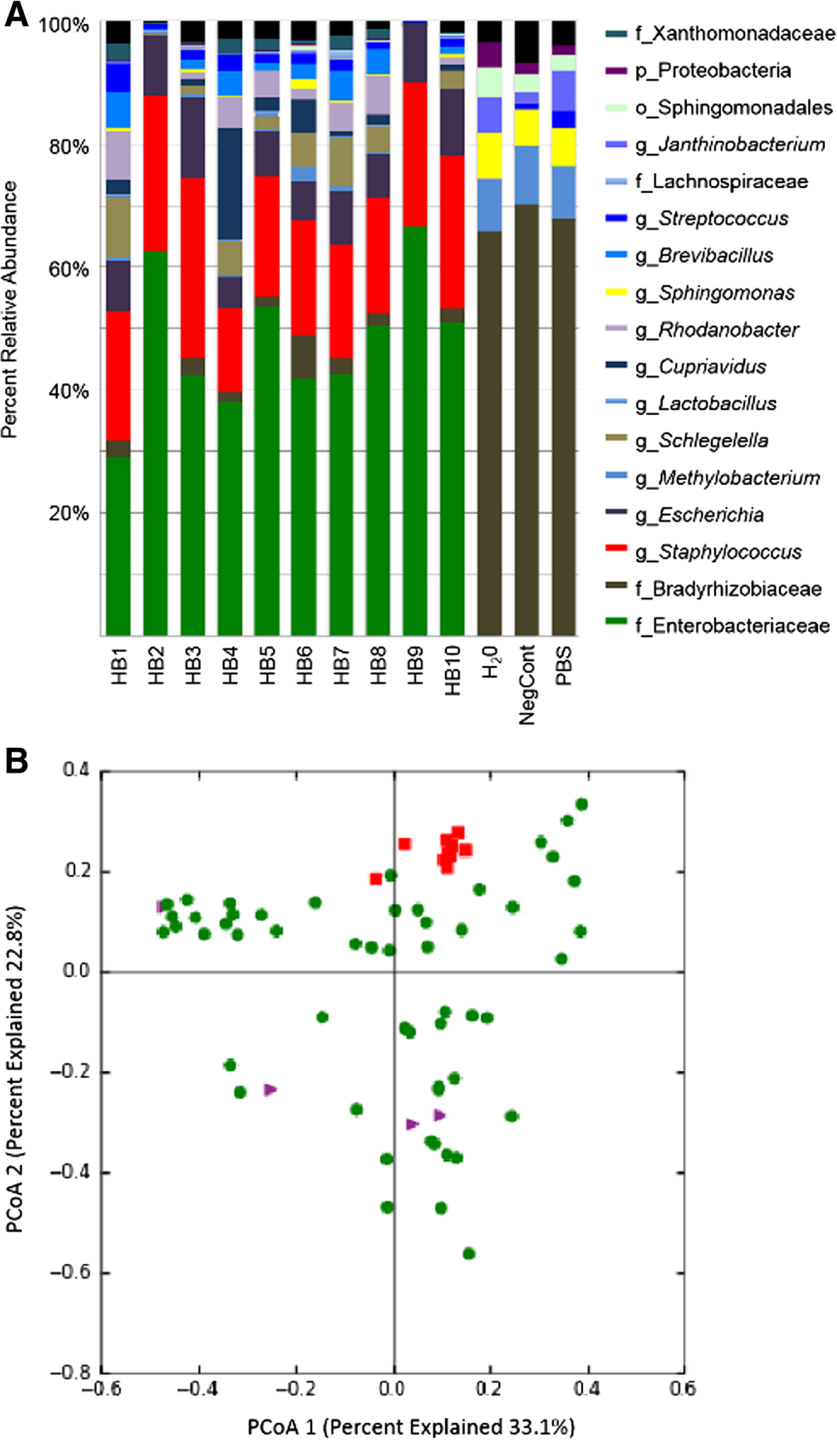
The bacteria DNA profiles of healthy blood. Whole blood was collected from 10 adult donors that worked in a health care setting but were healthy at the time of sampling. Two negative template controls (NTC) and sterile PBS were included for comparison. Taxonomic summaries for the blood samples were compared. Each bar represents the total DNA sequenced for the sample and the percent relative abundance of each OTU identified (**a**). The bacterial DNA profile profiles of the HB samples were similar to each other but distinct from the NTCs and PBS samples. The letter in front of each taxonomic group indicates the level of taxonomic depth with p__ representing phyla, f __representing family, o__ representing order, and g__ representing genus (**a**). Principal coordinates analysis (PCoA), based on weighted UniFrac, indicated the healthy blood samples (*red*) clustered separately from septic blood samples (*green*) (**b**)

In order to parallel the sepsis population cohort, whole blood from 12 healthy adults (age 38–73 years, median 43 years) was subjected to the same DNA extraction and sequencing protocols. The rationale was to determine if there was bacterial DNA in healthy donor blood or determine if there was a source of contamination in the processing of whole blood and DNA extraction. Of these 12 healthy donor blood samples, one failed to amplify in the initial PCR and one had less than 50 sequences amplified and were removed from further analysis. The remaining 10 samples had a minimum sequencing depth of 772 sequences per sample and a maximum of 33,133 sequences per sample with a median of 6174 sequences per sample. A total of 285,067 sequences were identified and aligned to 519 OTUs representing 105 taxonomic groups. In addition, PCR was performed on all the reagents and buffers used in the saponin blood-treatment and the DNA extraction. Of these, only PBS gave a positive signal and was included in the analysis as well as two negative template controls (NTC). The DNA amplified from the 10 healthy controls had similar taxonomic profiles (Fig. 5a). In all samples, the top OTUs were *Enterobacteriaceae, Staphylococcus,* and *Escherichia* representing 54.7 % up to 96.3 % of the OTU diversity in each sample. Lower levels of *Streptococcus* DNA were also present and ranged from 0.32 % to 4.17 % (Fig. 5a). The majority of the remaining OTUs did not represent human-associated bacteria.

Since the taxonomic identify of the OTUs in the health samples could also represent potential pathogens in a clinical setting, these taxonomic profiles were compared to a large cohort of whole blood samples collected from ICU patients. The results of the septic patient cohort will be discussed in a separate manuscript. Principal coordinates analysis (PCoA) of weighted unifrac metrics [28–30] was used to visualize the relationships between samples and indicated that the HB samples clustered separately from the sepsis samples (Fig. 5b). Permutational analysis of variance (PERMANOVA) was done to determine if these differences were statistically significant. This test was chosen, as it assumes no distribution and allows for the comparison of categorical factors such as sample type [31]. The PERMANOVA analyses supported the PCoA demonstrating that the healthy blood controls had taxonomic profiles that were significantly different from those seen in septic patients (*p* = 0.001).

### Case studies applying the saponin treatment to whole blood from septic ICU patients

**Fig. 6:**
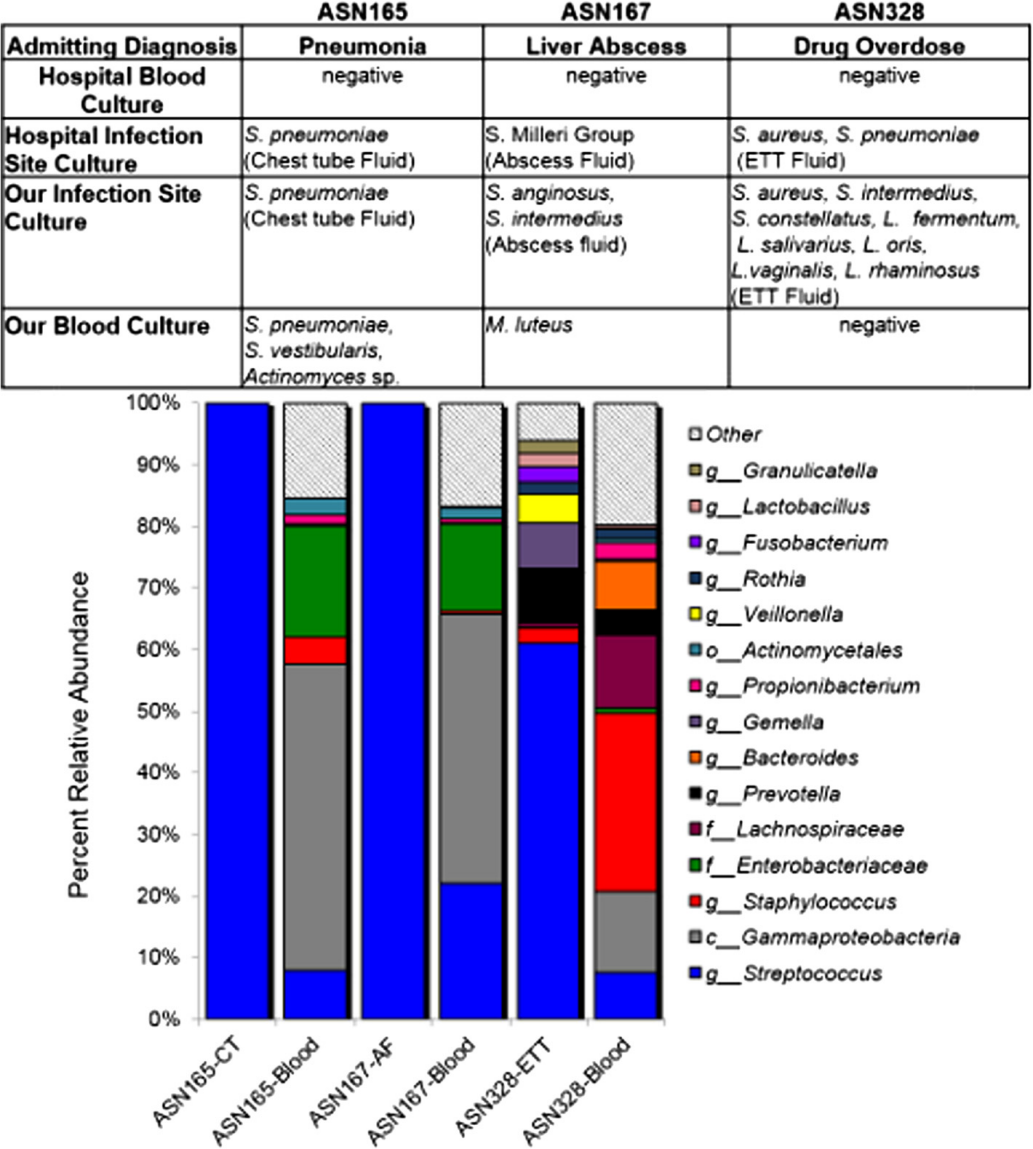
Bacterial DNA profiles of case studies from septic ICU patients. Taxonomic summaries of the bacterial DNA extracted from the primary infection sample and saponin treated whole blood from each case study patient. Each bar represents the combined results from two separate experiments with each PCR sample amplified in triplicate. The size of the bar indicated the percent relative abundance with the taxonomic identification labeled for each major group. The letter in front of each taxonomic group indicates the level of taxonomic depth with p__ representing phyla, f __representing family, o__ representing order, and g__ representing genus. The table indicated the comparison of the clinical diagnostic culture results to our culture results

Having established the method, we next set out to apply it to clinical samples collected from adult ICU patients. Three septic patient samples were characterized to evaluate how the bacterial DNA profiles could be interpreted in a clinical context. The first case involved a septic pneumonia patient, ASN165. Chest tube (CT) aspirate fluid was collected on Day 1 and 3 and whole blood was collected on Day 3 of the patient’s ICU stay. In-depth culture of the CT fluid recovered *S. pneumoniae* at 10^5^ CFU/ml (Fig. 6). Illumina sequencing of the 16S rRNA V3 region resulted in over 155,000 reads for Day 1 and Day 3 CT fluid. The genera *Streptococcus* represented 99.99 % OTU abundance on Day 1 and Day 3 (Fig. 6, Day 1 not shown)*.* The OTU representative sequence for the most prevalent *Streptococcus* OTU showed alignments to the *S. mitis/pneumoniae* group 16S rRNA. Day 3 whole blood was treated with saponin prior to in-depth culture. Partial 16S rRNA sequencing of recovered isolates indicated *Streptococcus* species, *S. vestibularis* and *Actinomycetes* species were present at less than 10 CFU/ml (Fig. 6). Molecular profiling of saponin treated whole blood collected on Day 3 resulted in 431 reads representing 10 OTUs at greater than 1 % of the relative DNA abundance. Diagnostic lab results for the blood culture results were negative on Day 1 and Day 3. Sputum culture indicated growth of *S. pneumoniae* on Day 1.

The second case study, ASN167, was a 37-year-old patient admitted to ICU with a pyogenic liver abscess related sepsis. Abscess drainage fluid and whole blood were obtained. In-depth culture of the abscess fluid indicated two members of the *Streptococcus anginosus*/*milleri* group (SMG); *S. anginosus* and *S. intermedius*, were present at 10^5^ CFU/ml (Fig. 6). Clinical lab culture of the abscess fluid also indicated heavy SMG growth. Molecular profiling of abscess fluid identified the genera *Streptococcus* representing 99.99 % of the total OTU abundance (Fig. 6). The OTU representative sequence for the most prevalent *Streptococcus* OTU showed alignments to the SMG group 16S rRNA. Saponin treated whole blood was cultured and *Micrococcus luteus* was recovered at less than 10 CFU/ml. Molecular profiling of saponin treated blood from ASN167 was done for Day 1 of ICU admission (Fig. 6). There were 4434 reads that matched to 9 OTUs above 1 % of the relative DNA abundance. Clinical laboratory blood culture results for ASN167 were positive for *Streptococcus milleri* Group three days prior to ICU admission. Upon ICU admission, blood culture results remained negative after 72 h of cultivation and may reflect rapid response to antibiotic therapy.

The final case study was ASN328, a 26-year-old male patient admitted to ICU with a drug overdose and suspected aspirtation pneumonia. In-depth culture of the ETT fluid indicated the patient had *S. aureus* at 10^3^ CFU/ml, *S. intermedius* and *S. constellatus* at 10^2^ CFU/ml, as well as five species of *Lactobacillus* (*L. fermentum, salivarius, oris, vaginalis, rhamnosus*) at 10^2^ CFU/ml. The diagnostic lab report indicated *S. aureus* present moderately and heavy *S. pneumonia*e. Molecular profiling of the ET fluid resulted in 10 OTUs at 1 % or higher relative DNA abundance. The most abundant OTU matched to the genera *Streptococcus* that represented 64.3 % of the relative DNA diversity (Fig. 6). The OTU representative sequence for the most prevalent *Streptococcus* OTUs showed alignments to the *S. salivarius/vestibulrius* group (25.5 %), the *S. mitis/pneumonia* group (23.7 %), and the SMG group (11.9 %). Molecular profiling of whole blood from ASN328 resulted in 3003 reads that fell into 16 OTUs representing 1 % or greater of the relative DNA diversity (Fig. 6). Sequences match to the genera *Staphylococcus* were the most abundant at 30.7 % (Fig. 6). Blood culture obtained from ASN328 on the same day was negative.

## Discussion

Currently, there is no “gold-standard” for DNA extraction from clinical samples. Many molecular studies have chosen DNA extraction methods without proper validation or rationale and used extraction protocols based on commercially available kits [6, 32–39] that have not been carefully evaluated in clinical samples [40]. As such, we developed our own comprehensive DNA extraction protocol for this study.

Our study demonstrated that the addition of the two enzymatic lysis agents, lysozyme and mutanolysin, optimized the recovery of polymicrobial DNA (Fig. 2). These results were comparable to those of Yuan *et al.*, 2012 that also found that a lytic enzyme cocktail of containing lysozyme, mutanolysin and lysostaphin (a *Staphylococcal* specific pentaglycin cleaving enzyme) consistently lysed cells of different species more effectively than lysozyme alone [40]. The addition of lysostaphin was deemed unnecessary given the results of the mock community demonstrated that *Staphylococcus* DNA was recovered at high abundance under all conditions (Fig. 3). The DNA extraction process included the addition of Proteinase K and RNase A to eliminate any PCR inhibitors present in the preparations and separate the DNA from organic components [40, 41]. In the final portion of the DNA extraction a column-based purification was employed to remove any remaining contaminants from the DNA preparation.

Our method used saponin at a concentration of 0.85 % mixed directly with 1-2 ml of whole blood. The use of detergents in sepsis diagnostics has been evaluated for blood culture media since the 1990s when it was shown to improve the recovery of fungal organisms, coagulase-negative *Staphylococcus* and *Pseudomonas* species while reducing the incidence of false-positive results [42]. Saponin interacts with cellular membrane components including phospholipids and sterols thereby resulting in the lysis of both red and white blood cells [19, 20]. In accordance with previous studies, no significant loss of bacterial growth across a panel of bacteria ranging from highly ubiquitous organisms (*E. coli, S. epidermidis*), fastidious organisms (*S. intermedius, pneumoniae*), and anaerobic organisms (*P. melaninogenica* and *F. necrophorum,* Fig. 1) was observed [19–22]. TRFLP results of the culture pools spiked into whole blood subsequently treated with saponin prior to DNA extraction suggested that our DNA extraction protocol could recover polymicrobial DNA from saponin-treated whole blood. There were limitations to these results that restricted our ability to assess microbial recovery. Namely, the TRFLP data only provided a semi-quantitative assessment and the culture pools were highly concentrated. As such, mock community of organisms recovered from sepsis infections (Table 1) was used in subsequent experiments assessing the applicability of our method in a cultivation-dependent as well as cultivation-independent approach.

Our results indicated a concentration dependent effect on the recovery of bacteria (Table 3). Gram-negative bacteria *E. coli* and *N. flava* had the poorest recovery in the mixed communities with higher inoculum required to recovery these organisms compared to the other bacteria (Table 3). Other culture-independent studies have reported under-representation with Gram-negatives including *E. coli* for unknown reasons [40]. In order to determine if there was a competitive effect, each organism was spiked individually into blood and the recovery was assessed (Fig. 3). The same phenomenon was observed with 1–2 log drops in *E. coli* and 3–4 log drops in *N. flava* CFU following treatment. Our findings suggest the inhibition of growth was likely due to bacteriostatic and bactericidal effects of human blood cells and plasma [43]. The use of sodium polyanethole sulfonate (SPS) in clinical culture media is often used to circumvent the bacteriostatic/bactericidal effects [43]. SPS is also a strong PCR inhibitor [41] and we were unable to use SPS-treated samples for molecular profiling (data not shown). Overall, the recovery of a panel of organisms spiked into whole following the saponin treatment with or without hypotonic washes suggested that saponin treatment could be used to recover bacterial cells directly from whole blood without compromising microbial viability.

Illumina based profiling of the DNA recovered from the mock communities indicated the saponin lysis followed by two washes with DNase/RNase free water resulted in the most representative bacterial DNA profile (Fig. 4). All of the mock community organisms were identified but at varying levels of sequence resolution ranging from *M. luteus* identified as *Actinomycetales,* the *E. hormaechei* identified as *Enterobacteriaceae,* and the remaining organisms identified at the genera level (Table 4). The composition of the bacterial DNA profiles paralleled the culture-based composition.

In developing a molecular profiling strategy, there were several limitations that needed to be addressed. The first was the primer cross-reactivity with human DNA. Non-specific amplification of human DNA with universal 16S primers has been well documented [26, 27, 44, 45]. However, the abundance of human DNA often represented a large portion of the amplified sequences in whole blood likely reflecting the low ratio of bacterial to host DNA in these samples. The proportion of amplified human DNA increased as the concentration of bacteria decreased. Nevertheless, these sequences were easily removed from the taxonomic profile thereby permitting analysis of the bacterial components of the DNA profiles. The use of other 16S rRNA primer sets was considered but the paired-end Illumina V3 region was selected as it had better taxonomic resolution and longer length when compared to other regions [46, 47].

The bacterial DNA amplified from healthy control samples was not unexpected as our PCR approach was exquisitely sensitive and the universal 16S primers allow for the amplification of any DNA from a bacterial source [48]. Contamination from reagents and the environment (laboratory and hospital) is a common problem in PCR using universal bacterial gene probes. The bacterial DNA in the taxonomic profiles from the control samples were represented by OTUs for *Enterobacteriaceae, Escherichia,* and *Staphylococcus* (Fig. 5a). These OTUs were not recovered from the NTS or PBS control samples suggesting PCR reagents were likely not the source of this bacterial DNA (Fig. 5a). The DNA present in these samples was interpreted as a mixture skin and environment contamination as a result of the way in which the blood samples were collected. The samples were peripheral blood draws into vacutainers. Since this was not done in a hospital setting, the same procedures used for blood culture to minimize skin contamination were not employed [49]. Further, studies have indicated that antisepsis at the skin cannot completely prevent contamination since 20 % of the skin bacteria are located in deep layers of the skin or in structures that the surface antiseptics do not penetrate [49]. In comparison, our septic patient blood samples were collected mainly from central lines. Further, the skin was clean using a sterile isopropyl alcohol swab. These swabs are effective at killing bacteria but do not degrade bacterial DNA [50]. As such, DNA from skin microbiota may have been introduced during the venipuncture of the skin. This could account for the abundance of *Staphylococcus* DNA recovered as the *Staphylococcus* are considered as part of the skin microbiota [51]. Although the *Enterobacteriaceae* are not commonly associated with the skin microbiota, a study assessing the hands of health care workers recovered *Enterobacter* species as well as other Gram-negative bacteria, not taxonomically identified, in addition to *Staphylococcus* species from individuals in which there was documented skin damage [52]. Overall, the DNA representing human-associated taxonomic groups was interpreted as representative of skin microbiota contaminating the venipuncture. We also compared the ten healthy control samples to a cohort of blood samples collected from 62 ICU patients with sepsis. The taxonomic profiles of the septic patient blood samples clustered separately and were statistically different from all the healthy control samples (Fig. 5b). Attempts to quantitate the bacterial load in the healthy control samples, using Real time-PCR, were unsuccessful due to the cross-reactivity of the 16S primers to human DNA in these samples (data not shown). As such, the total abundance of the bacterial DNA in the healthy control samples was unknown. Nevertheless, knowledge of this type of contamination in the whole blood samples indicated that caution was needed for the interpretation of the *Staphylococcus, Enterobacteriaceae,* and *Escherichia* OTUs present in the clinical sample’s molecular profiles. These OTUs were not interpreted as significant unless there was clinical evidence to support the presence of bacterial DNA represented by these OTUs in the patients.

In the mock community samples, the PCR amplified DNA was distributed into 203 OTUs sequenced thereby overestimating the diversity in these mock communities consisting of 8 bacterial genera. The over-estimation of diversity in next-generation sequencing studies has been well documented [53]. In particular, the use of heuristic methods for OTU clustering overestimates the number of groups. With our methods optimized to recover all microbial DNA, there was also a risk of amplifying any environmental DNA present during the blood collection, the DNA extraction procedure, and the PCR set-up. Other studies have also reported recovery of DNA not correlated to the mock community, and found higher rates of contamination in samples with lower bacterial DNA [54]. Our study exhibited the same phenomenon as the mock communities containing the lowest CFU/ml of bacteria resulted in the greatest abundance of OTUs that could not be correlated to the original mock community. Although there was still over-estimation of diversity, the mean proportion of OTUs that could not be taxonomically assigned to the mock community organisms was 26.7 %. These OTUs were considered contaminant DNA that were minor components of the taxonomic profile yet when the template DNA became limiting, their relative abundance was increased. For future clinical diagnostic application it will be important to minimize contamination from reagents.

Despite some limitations in the mock communities, the Illumina molecular profiling of saponin treated blood was successfully applied to three ICU case studies. In the case of ASN165, the molecular profiling data indicated that this patient had *S. pneumoniae* pneumonia that progressed to a bloodstream infection (Fig. 6). The culturing of saponin treated blood and the molecular profiling method provided supporting evidence for a *S. pneumoniae* infection with a positive blood culture and a dominant *Streptococcus* OTU alignment to the *S. mitis/pneumonia* group. The clinical diagnostic laboratory blood culture was negative for any organism despite our recovery of *Streptococcus* bacteria and DNA from blood collected the same day as the blood culture. For patient ASN167, the clinical data suggested that the patient no longer had bacteremia, however, the molecular profiling data suggested *Streptococcus* bacteria or bacterial products were still present in the bloodstream (Fig. 6). This would not be unexpected as abscess formation provides a reservoir of bacteria that can continuously be shed into the bloodstream [9]. The molecular profiling data indicated that the patient still had detectable *Streptococcus* DNA signal despite a negative clinical diagnostic blood culture. For ASN328 the molecular profiling data and in-depth culture data suggested a more complex infection aetiology when compared to the clinical diagnostic results. The presence of several *Lactobacillus* species, *Prevotella,* and *Fusobacterium* in the ETT fluid from this patient suggested that there was an aspiration event in addition to the *Streptococcus* and *Staphylococcus* infection (Fig. 6). Molecular profiling also detected *Rothia, Prevotella, Granulicatella, Veillonella, Gemella*, and *Fusobacterium* OTUs that have also been recovered in studies on chronic airway infections [24, 25]. The blood molecular profile for ASN328 shared several OTUs with the ETT fluid (Fig. 5) suggesting that there was possible contribution of these bacteria or their DNA to a bloodstream infection despite the negative blood culture results.

Polymicrobial DNA profiles were identified in all three patients using a non-targeted molecular profiling methods. In addition, some of the OTUs identified in the blood molecular profile were the same genus as bacteria cultivated from either the primary infection sample or the blood sample (Fig. 6). There were varying levels of sequencing depth between samples despite normalizing the DNA concentration used in the PCR reactions. This may reflect varying levels of bacterial DNA template present in each sample. The mock community analysis suggested that as the level of bacteremia decreased the proportion of amplified human DNA indicating that the issue was associated with the ratio of bacterial to human DNA. As such, samples with lower number of reads reflected a higher ratio of human to bacterial DNA. Since this was a ratio-based issue, the use of larger blood volumes was not predicted to circumvent these limitations. Nevertheless, the removal of these DNA sequences from the taxonomic profile enabled the analysis of the remaining, low proportion, bacterial DNA in the case study samples. Based on this preliminary data, we concluded that the use of a saponin-mediated blood cells lysis combined with a robust DNA extraction method could be used to enable the successful application of an Illumina molecular profiling approach to identify bacterial infection in the blood and primary infection samples of sepsis patients.

## Conclusions

The paired-end Illumina 16S rRNA community profiling of bacteria has been successfully applied to human clinical samples to provide a more robust evaluation of polymicrobial infections [47, 55–57]. To our knowledge, this was the first study in which paired-end Illumina 16S rRNA gene community profiling was done on whole blood. The results of this study indicate that a saponin blood pre-treatment lysis steps combined with the paired-end Illumina sequencing enabled molecular-profiling of small volumes of whole blood. Blood culture based assessments indicate that the incidence of polymicrobial sepsis is low ranging from 10–20 % [17, 58]. However, the limited results from these case studies suggest that a molecular approach may enable improved detection of polymicrobial infections. The application of sensitive molecular methods to clinical samples can identify more organisms in samples when compared to clinical diagnostics, which is selective for specific organisms. It is important to consider that DNA based methods indicate the presence of DNA rather than viable organisms and positive results should be interpreted accordingly. Whether or not they represent viable organisms in the blood, a positive signal indicates the presence of bacterial products in the blood, which would contribute to systemic inflammation. Future work will involve application of this protocol to subsets of adult and paediatric sepsis patients to further verify the utility of this method.

## Methods

### Isolates and culture conditions

Isolates were obtained from clinical specimens as outlined in Table 1. Isolates were maintained at -80C in 10 % skim milk for long term storage. Samples were grown on solid media including Columbia blood agar (CBA), Colombia CNA agar (CNA), mannitol salt agar (MSA), and trypticase soy agar supplemented with 3 % yeast extract (TSY), all from BD Diagnostics, Canada as well as, fastidious anaerobic agar (FAA, Neogen Acumedia, USA). Difibrinated sheep’s blood (Dalynn Biologics, Canada) was added to a 5 % final volume for CBA, CNA, and FAA. Isolates were cultivated at 37 °C with 5 % CO_2_ or in a Ruskinn Concept 400 anaerobic chamber (Ruskinn, Bridgend, UK) with 80 % N, 10 % CO_2_ 10 % H_2_.

### Sample collection and processing

Approval for this study was obtained from the Conjoint Health Research Ethics Board of the University of Calgary. All human samples were collected following the guidelines outlined in the Canadian of Health Research, Natural Sciences and Engineering Research Council of Canada, and Social Sciences and Humanities Research Council of Canada, Tri-Council Policy Statement: Ethical Conduct for Research Involving Humans, December 2010. Written informed consent was obtained for all patients, or their proxy decision maker, enrolled in the study. Patients of 18 years of age or older admitted to the ICU of the Foothills Medical Center meeting the published criteria for systemic inflammatory response and clinical suspicion of sepsis within the first 24 h of ICU admission were eligible for the study [1, 59, 60]. Clinical and laboratory data was collected daily during the ICU stay of the patient and mortality was tracked until hospital discharge. Control individuals were adults above 30 years of age who worked in a health care setting but at the time collected had no major autoimmune disorders, no symptoms of illness, were not on anti-inflammatories for the prior 7 days, and were otherwise healthy (no colds/fever/chills/respiratory symptoms) in the prior 7 days with a normal respiratory rate and temperature measured as supportive data. Collection was done as part of the Critical Care Epidemiologic and Biologic Tissue Resource (CCEPTR). Approval for CCEPTR was granted by the Conjoint Health Research Ethics Board of the University of Calgary with the Ethics ID for the study E-22236 on April 7, 2009. Whole blood was collected from an existing arterial line, central line or venous line with a maximum volume of 4 ml in sterile K_2_EDTA spray coated vacutainers (BD Diagnostics). Biologic samples included bronchoalveolar lavage (BAL) fluid, endotracheal tube (ETT) fluid and abscess drainage fluid (AF) collected in sterile containers.

Biological fluid samples were first sheared to an even consistency using a 1 ml tuberculin slip-tip syringe. Ten-fold serial dilutions were prepared as needed. Samples were plated using 100 μl of sample per solid media type and incubated as indicated above.

### Bacterial viability with Saponin

To evaluate whether saponin impacted bacterial viability, *in vitro* experiments were carried out in which bacterial strains recovered from sepsis infections (Table 1) were co-incubated with 0.85 % saponin at concentrations ranging from 10^1^ to 10^9^ CFU/ml for 60 min. Whole blood was treated with 0.85 % saponin (Sigma-Aldrich, USA) final volume at room temperature for 15 min to lyse red blood cells. Following treatment, the blood was centrifuged at 20,800 rcf for 15 min to remove lysed cells in the supernatant. The supernatant was removed and the remaining cells were washed 1-3x with 1 ml sterile DNase/RNase free double distilled water (Life Technologies, Burlington, ON, Burlington, ON). This involved re-suspending the pellet in the sterile water and vortexing to ensure homogeneity prior to centrifugation. After each wash the cells were spun at 20,800 rcf for 15 min and the supernatant was removed. Cells were suspended in 500 μl sterile PBS for storage prior to DNA extraction or in 500 μl BHI broth for cultivation.

### DNA extraction from whole blood

To each 500 μl sample of saponin treated blood, 50 μl of Lysozyme (100 mg/ml, Sigma-Aldrich, Oakville, ON), 20 μl of Mutanolysin (10U/μl, Sigma-Aldrich, Oakville, ON), and 20 μl of RNase A (10 mg/ml, Life Technologies, Burlington, ON) were added and incubated overnight at 37 °C. Following this, 50 μl of 25 % sodium dodecyl sulphate (SDS, Sigma-Aldrich, Oakville, ON), 50 μl of 20 mg/ml proteinase K (Invitrogen, Life Technologies, Burlington, ON), and 100 μl 5 M NaCl were added. The mixture was incubated at 65 °C for 1–2 h. Cellular debris was pelleted by centrifugation at 20,800 rcf for 10 min. The supernatant was then treated with one standard phenol-chloroform-isoamyl (25:24:1, Life Technologies, Burlington, ON) alcohol extraction. The DNA in the aqueous layer was transferred to a Zymo DNA Clean & Concentrator™-25 (Zymo Research, Irvine, CA) column containing 200 μl of ChIP DNA Binding Buffer (Zymo Research, Irvine, CA). The column was spun for 1 min at 20,800 rcf and the flow-through was discarded. Wash buffer was added at 500 μl twice to the column with a 1 min, 20,800 rcf centrifugation and discard of flow-through in between each wash. A final 1 min, 20,800 rcf centrifugation step was done to ensure the column was completely dry and free of any ethanol carry-over from the wash buffer. Pre-warmed DNase/RNase free deionized and UV irradiated water was used to elute the DNA with 50 μl added per column. DNA was quantified using a Nanodrop 2000c Spectrophotometer.

### Optimization of DNA extraction and purification

In order to validate the DNA extraction, whole blood was collected in K_2_EDTA vacutainers and spiked with a mixed bacterial community recovered on CBA from a septic pneumonia BAL fluid sample from an ICU patient (Table 2). This plate pool represented bacteria recovered at or greater than 10^3^ CFU/ml. All bacteria recovered on the CBA plate were resuspended in 1 ml of sterile 10 % skim milk and stored at −80 °C. Aliquots of 500 μl of blood were spiked with 50 μl of the culture pool and subjected to a 0.85 % saponin treatment. DNA was extracted from the resulting blood pellet in one of three ways; with no enzymatic lysis, lysozyme alone, and lysozyme plus mutanolysin. The RNAase and proteinase K treatments were the same for all three samples. The supernatant was then treated with one standard phenol-chloroform-isoamyl (25:24:1, Life Technologies, Burlington, ON) alcohol extraction. The DNA in the aqueous layer was transferred to a Zymo DNA Clean & Concentrator™-25 (Zymo Research, Irvine, CA) column containing 200 μl of ChIP DNA Binding Buffer (Zymo Research, Irvine, CA). The column was spun for 1 min at 20,800 rcf and the flow-through was discarded. Wash buffer was added at 500 μl twice to the column with a 1 min, 20,800 rcf centrifugation and discard of flow-through in between each wash. A final 1 min, 20,800 rcf centrifugation step was done to ensure the column was completely dry and free of any ethanol carry-over from the wash buffer. Pre-warmed DNase/RNase free deionized and UV irradiated water was used to elute the DNA with 50 μl added per column. DNA was quantified using a Nanodrop 2000c Spectrophotometer.

### Partial 16S rDNA sequencing

Grown colonies of bacteria were distinguished using colony morphology and identification was done on 10 % chelex (Bio-Rad, USA) boiled colony preps [61] using PCR with the universal 16S rDNA primers 8 F (5'AGAGTTTGATCCTGGCTCAG3') and 926R (5'CCGTCAATTCCTTTRAGTTT3'). PCR cycle conditions were initial denaturation at 94 °C for 1 min, 32 cycles of denaturation at 94 °C for 1 min, annealing for 1 min at 57 °C and extension at 72 °C for 1 min followed with a final extension at 72 °C for 10 min. PCR products were sequenced unidirectionally from the 8 F primer at Beckman Coulter Genomics (Danvers, USA) and the sequences were aligned to curated ribosomal sequence databases including HOMD (human oral microbiome database, www.homd.org), and Greengenes (greengenes.lbl.gov/cgi-bin/nph-index.cgi).

### Terminal restriction fragment length polymorphism

Molecular analysis was done using terminal restriction fragment length polymorphism (TRFLP) as outlined by Sibley et al. [25]. Briefly, a portion of the 16S rRNA gene was PCR amplified using 8 F (5'AGAGTTTGATCCTGGCTCAG3') and 926r (5'CCGTCAATTCCTTTRAGTTT3'). The 8 F primer was fluorescently tagged with 6-FAM at the 5′ end. PCR conditions were the same as those outlined above. PCR products were concentrated and purified using Zymo DNA Clean and Concentrator™ columns (Zymo Research) then digested overnight with CfoI (10U, Sigma-Alrdich). Fragment analysis was done at the UCDNA core facility (University of Calgary, Canada). Analysis was done using GeneMapper 4.0 (Applied Biosystems, Life Technologies) and the percent of total peak area for each fragment size was calculated.

### 16S rRNA gene bacterial community profiling with paired-end Illumina

PCR and sequencing was done following Bartram *et al.* (2011) with some modifications. PCR amplification of the V3 region was done using the primers 341 F (5′CCTACGGGAGGCAGCAG3′) and 518R (5′ATTACCGCGGCTGCTGG3′). Primer modifications included the addition of Illumina multiplexing, bridge amplification and sequencing regions [47]. Reverse primers were barcoded to allow multiple processing of samples. For whole blood samples, 200–300 ng of DNA was PCR amplified whereas 50-100 ng of DNA was used for primary infection site samples. The PCR cycle consisted of an initial denaturation at 94 °C for 2 min followed by 30 cycles of denaturation at 94 °C for 30 s, annealing at 50 °C for 30 s, extension at 72 °C for 30 s followed by a final extension at 72 °C for 10 min. Each sample was amplified in triplicate and the pooled PCR reactions were run on a 2 % agarose gel. PCR products were excised from the gel and purified using the QIAquick gel extraction kit (Qiagen, Netherlands) following manufacturer guidelines. The resulting PCR products were sequenced using the Illumina MiSeq personal sequencer (Illumina Incorporated, USA) at the McMaster Genomics Facility, Ontario, Canada. After sequencing, image analysis, base calling, and error estimation were completed using the Illumina Analysis Pipeline (version 2.6) [47]. The sequencing data was processed with custom, in-house Perl scripts [62]. Initial sequence processing was carried out with Cutadapt [63] and paired-end sequences aligned and consensus generated using PANDAseq [64]. Operational taxonomic units (OTUs) clustering at a threshold of 97 % sequence similarity was carried out using AbundantOTU+ [65]. The taxonomic identification was assigned using the Ribosomal Database Project classifier [66] using the Greengenes reference database, February 4^th^ 2011 release [67] as a training set. QIIME computational analysis pipeline was used for community analysis (alpha and beta diversity) [68]. β-diversity was used as a measure to examine differences between samples. Both weighted and un-weighted UniFrac distance and clustering of the samples was done and visualised using principal coordinate analysis (PCoA) [28, 30]. PCoA plots were visualized using KiNG version 2.21 visualization software [69].

### Synthetic community experiments

Ten bacterial strains recovered from sepsis cases (bloodstream or organ site infections (Table 1) were used to generate a mixed community. Colonies from each sample were suspended in sterile M9 salts + 0.1 % cysteine to a McFarland 2 unit. Viability in saponin was assessed by incubating 10^−1^ through to 10^−8^ dilutions of each isolate with 0.85 % saponin for 1 h at room temperature. Colony forming units (CFU) were determined prior to and after treatment as an average of duplicate plates. Communities of 100 μl of each strain diluted at a 10^−1^, 10^−3^, or 10^−5^ dilution were mixed together resulting in the three communities SC1, SC3, and SC5 respectively. Human whole blood was collected in K_2_EDTA vacutainers and 20 μl of each synthetic community was added to 500 μl of whole blood. Saponin was added at a 0.85 % final volume and incubated for 30 min at room temperature. Three treatments with saponin were performed; addition of 0.85 % saponin with no further washes, addition of saponin at 0.85 % with the addition of a 1 ml sterile double distilled water wash, or addition of saponin at 0.85 % with two washes with 1 ml sterile double distilled water. All recovered cells were resuspended in BHI and plated in duplicate on MSA, CNA, MAC, TSY, and FAA using 100 μl per plate prior to and after addition to blood. In addition, 10 μl of each strain alone was diluted and spiked into 250 μl of 0.85 % saponin treated whole blood, incubated for 30 min at room temperature and plated on their respective media type (Table 1). DNA was also extracted from all communities for TRFLP and 16S rRNA gene community profiling. These experiments were done in triplicate with three different samples of human blood done on different days.

## Abbreviations

AF: Abscess fluid
BAL: Bronchoalveolar lavage
BHI: Brain-heart infusion
BHIco: Brain-heart infusion with colistin and oxolinic acid
BLAST: Basic local alignment search tool
CBA: Colombia blood agar
CCEPTR: Critical care epidemiologic and biologic tissue bank resource
CNA: Colombia CNA agar
CT: Chest tube fluid
ETT: Endotracheal tube fluid
FAA: Fastidious anaerobe agar
HOMD: Human oral microbiome database
MAC: MacConkey agar
MSA: Mannitol-salt agar
OTU: Operational taxonomic unit
QIIME: Quantitative insights into microbial ecology
SC: Synthetic community
SMG: *Streptococcus milleri* group
T-RF: Terminal restriction fragment
TRFLP: Terminal restriction fragment length polymorphism
TSY: Tryptic soy yeast
